# The Latin Network of Researchers in Reproductive Biotechnologies: scientific cooperation and social debate in a global era

**DOI:** 10.5935/1518-0557.20230003

**Published:** 2023

**Authors:** Rosana Machin, Consuelo Álvarez Plaza

**Affiliations:** 1 Universidade de São Paulo, Faculdade de Medicina, Departamento de Medicina Preventiva; 2 Complutense University of Madri, Anthropology Department, Spain

The development of assisted reproductive technologies has raised a number of issues to be debated in an interdisciplinary manner. Formulated for the treatment of fertility problems among heterosexual couples, its development enabled the access of other subjects to the construction of affiliation projects such as single women and men, male and female homosexual couples and transgender people. In this context, assisted reproductive technologies challenged traditional norms of reproduction, generating new family models.

In the first decades of assisted reproduction, discussions revolved around who should have access to the techniques and the right of those born to know their genetic origins, as well as the implications of breaking anonymity. However, a series of new questions instigate the scientific and social debate about reproductive mobility and cross-border reproduction, reproductive work, the participation of third parties in affiliation projects through the donation of genetic material or via surrogacy, the risks involved in the donation of genetic material, the adoption of embryos, the quota of births by donation of genetic material and the persistence of problems linked to socioeconomic inequalities in access to technology. The expansion of reproductive technologies encourages discussions on ethical and legal aspects involved in topics such as affiliation, territory, nationality and reproductive autonomy, updating issues related to social, ethnic, racial and gender inequalities.

Based on the need to reflect on this context, we created the Latin Network of Researchers in Reproductive Biotechnologies in 2019 (Redlibre, 2022), from a cooperation project supported by the Iberoamerican Union of Universities (UIU). The UIU is a strategic alliance formed by the University of Sao Paulo (USP), University of Barcelona (UB), University of Buenos Aires (UBA), Autonomous University of Mexico (UNAM) and Complutense University of Madrid (UCM).

The “Latin” in our name indicates the participation of researchers of Latin origin and interest in the subject, who also share the importance of the Catholic cultural tradition and its discussion in this field. The Catholic cultural tradition, for centuries configured a way of thinking about the world in the countries where its presence was widespread. This presence was not limited to the religious context, but influenced the legal, social, cultural and state business fields. Catholic culture sought to influence and dictate rules of morality in relationships surrounding family, gender and kinship. Although the weight of this tradition has decreased in many countries, it has left its marks on the ways in which the meanings attributed to affiliation, family, blood ties and maternity/paternity are understood. The Catholic cultural tradition is not our object of reflection directly, but it sets precedence for discussing practices in assisted reproductive technologies.

The network (Redlibre, 2022) has grown since 2019 and currently brings together more than forty (40) researchers from different fields of knowledge (Anthropology, Biology, Law, Medicine, Philosophy, Psychology and Sociology) specialized in reproductive biotechnologies from several universities in Spain, Italy, Belgium, Germany, Switzerland, Argentina, Brazil, Chile, Mexico and Uruguay.

Our objective is to reflect on the impact of reproductive technologies among its users, as well as the offspring generated by them, identifying the main challenges posed. In this regard, we have sought to strengthen scientific cooperation between our institutions, disseminate knowledge that is produced, and expand the social debate on technologies in assisted reproduction through workshops and monthly international seminars. In these seminars, we discuss topics such as surrogacy in conflict situations (with the participation of a pregnant woman from Ukraine), the reproduction market in Spain, the issue of anonymity, with the participation of adults born through gamete donation, and racial matching between donors and receptors in reproductive clinics. Event registration can be accessed through our website. The network hopes to contribute and expand, in a qualified, way the social debate on assisted reproductive technologies.
